# Batf3-dependent orchestration of the robust Th1 responses and fungal control during cryptococcal infection, the role of cDC1

**DOI:** 10.1128/mbio.02853-23

**Published:** 2024-02-13

**Authors:** Jintao Xu, Rylan Hissong, Rachel Bareis, Arianna Creech, Kristie D. Goughenour, Christine M. Freeman, Michal A. Olszewski

**Affiliations:** 1 Research Service, Department of Veterans Affairs Health System, Ann Arbor VA Health System, Ann Arbor, Michigan, USA; 2 Division of Pulmonary and Critical Care Medicine, Department of Internal Medicine, University of Michigan Health System, Ann Arbor, Michigan, USA; Albert Einstein College of Medicine, Bronx, New York, USA

**Keywords:** dendritic cells, fungal infection, cryptococcal infection, Th1 responses, Batf3, cDC1

## Abstract

While type I conventional dendritic cells (cDC1s) are vital for generating adaptive immunity against intracellular pathogens and tumors, their role in defense against fungal pathogen *Cryptococcus neoformans* remains unclear. We investigated the role of the cDC1 subset in a fungus-restricting mouse model of cryptococcal infection. The cDC1 subset displayed a unique transcriptional signature with highly upregulated T-cell recruitment, polarization, and activation pathways compared to other DC subsets. Using Batf3^−/−^ mice, which lack the cDC1 population, our results support that Batf3-dependent cDC1s are pivotal for the development of the effective immune response against cryptococcal infection, particularly within the lung and brain. Deficiency in Batf3 cDC1 led to diminished CD4 accumulation and decreased IFNγ production across multiple organs, supporting that cDC1s are a major driver of potent Th1 responses during cryptococcal infection. Consistently, mice lacking Batf3-cDC1 demonstrated markedly diminished fungicidal activity and weaker containment of the fungal pathogen. In conclusion, Batf3-dependent cDC1 can function as a linchpin in mounting Th1 response, ensuring effective fungal control during cryptococcal infection. Harnessing cDC1 pathways may present a promising strategy for interventions against this pathogen.

**IMPORTANCE:**

*Cryptococcus neoformans* causes severe meningoencephalitis, accounting for an estimated 200,000 deaths each year. Central to mounting an effective defense against these infections is T-cell-mediated immunity, which is orchestrated by dendritic cells (DCs). The knowledge about the role of specific DC subsets in shaping anti-cryptococcal immunity is limited. Here, we demonstrate that Batf3 cDC1s are important drivers of protective Th1 CD4 T-cell responses required for clearance of cryptococcal infection. Deficiency of Batf3 cDC1 in the infected mice leads to significantly reduced Th1 response and exacerbated fungal growth to the point where depleting the remaining CD4 T cells no longer affects fungal burden. Unveiling this pivotal role of cDC1 in antifungal defense is likely to be important for the development of vaccines and therapies against life-threatening fungal pathogens.

## INTRODUCTION

The *Cryptococcus neoformans* species complex consists of fungal pathogens that present a significant challenge to global public health (1, 2). The most devastating disseminated cryptococcal disease results in meningoencephalitis, responsible for nearly 200,000 fatalities worldwide every year (1). Despite advances in antifungal treatments, the mortality rate associated with cryptococcal meningoencephalitis remains high (3, 4), underscoring the urgent need for a better understanding of host-pathogen interactions and the development of novel therapeutic strategies.

Successful clearance of cryptococcal infections hinges on T-cell immunity and its appropriate polarization (5, 6). The emergence of antigen-specific Th1, Th2, Th17, and regulatory T cells (Treg) determines the outcomes of the infection, largely due to the secretion of specific effector cytokines. Interferon (IFN)-γ, produced by Th1 cells, and interleukin (IL)-17, secreted by Th17 cells, contribute to the recruitment and activation of innate phagocytes, leading to protective immunity (7, 8). In contrast, enhanced production of Th2 or Treg-type cytokines (such as IL-4, IL-10, and IL-13) promotes alternative activation of macrophages, which facilitates intracellular survival and fungal growth in the host (9, 10).

Dendritic cells (DCs) are pivotal antigen-presenting cells that capture, process, and present pathogen-derived antigens to T cells through major histocompatibility complex (MHC) molecules (11, 12). DCs comprise a heterogeneous group with distinct phenotypic and functional attributes. These include conventional DC subsets like cDC1 (IRF8- and Batf3-dependent, marked by XCR1 and CD103 expression) and cDC2 (identified by SIRPa expression), as well as monocyte-derived DC that arise during inflammatory events (12, 13).

Recent studies have highlighted the importance of the basic leucine zipper ATF-like transcription factor 3 (Batf3)-dependent cDC1 in shaping cytotoxic T lymphocyte responses by their ability to cross-present antigens on MHC class I molecules (14–16). cDC1 also exhibits surface and endosomal pattern recognition receptors (PRRs) that are proficient at identifying pathogen-associated molecular patterns (PAMPs). Moreover, they are significant producers of IL-12 in some disease settings (17–19), a cytokine that promotes Th1 responses and is crucial for protection against cryptococcal infections (20). However, the knowledge about the role of Batf3 cDC1 in fungal infections is very limited. They have a significant role in defense against endemic *Histoplasma capsulatum* mycosis (21, 22) but are dispensable for mucosal and systemic defenses against candidiasis (21, 22). Considering the vital reliance on Th1 for cryptococcal clearance, we hypothesized that Batf3-dependent cDC1 is instrumental in driving protective immunity against cryptococcal infection.

Our study provides strong evidence that Batf3-dependent cDC1 promotes protective Th1 CD4 T-cell activities during cryptococcal infection. The absence of Batf3 cDC1 led to heightened fungal growth across various organs. These findings highlight the importance of Batf3 and cDC1 in anti-cryptococcal defenses and offer valuable insights for the development of targeted immunotherapies and vaccines against this fungal pathogen.

## RESULTS

### cDC1, in a Th1-dominant immune response to cryptococcal infection*,* displays a unique mRNA signature with highly pronounced transcripts for T-cell recruitment and polarization pathways

**Fig 1:**
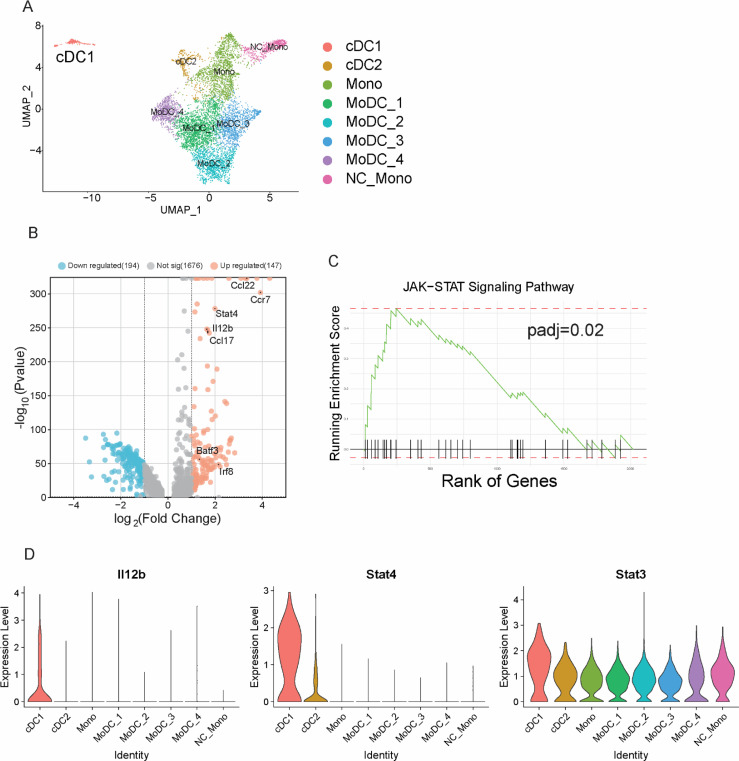
cDC1 gene expression patterns indicate a role in T-cell recruitment and polarization during cryptococcal infection. CD45+ immune cells from the brain on days 0, 7, 14, 21, and 28 post-infection were enriched and subjected to scRNA-seq analysis. Subsequent detailed analyses focused on DCs, monocytes, and their derived cells. (**A**) UMAP representation of brain DC and monocyte-derived cell populations. (**B**) Volcano plot showcasing differentially expressed genes in cDC1 relative to other DC and monocyte-derived cells. (**C**) Enrichment analysis for the JAK-STAT signaling pathway by cDC1 population. (**D**) Gene expression levels of *IL12b*, *Stat4*, and *Stat3* among different cell populations. The presented data were generated by pooling immune cells from three mice per time point. Pooled cells (~5,000 cells total per sample) were submitted for sequencing.

To assess DC diversity and function during protective anti-cryptococcal immunity we employed a murine model of disseminated cryptococcosis characterized by a dominant Th1 response and robust fungal clearance in multiple organs (23, 24). C57BL6/J mice were infected with cryptococcal strain 52D (ATCC 24067) intravenously. CD45+ immune cells were isolated from the infected brain at various time points and analyzed by single-cell RNA sequencing as described in Materials and Methods. UMAP analysis revealed unique cell sub-clusters among DC and monocyte-derived populations based on their gene expression profiles (Fig. 1A). Following the identification of conventional DC cells by their unique expression of Dpp4, Zbtb46, and Flt3 (Fig. 1A; Fig. S1A), we distinguished clusters of cDC1 (marked by *Irf8*, *Ccr7*, and *Ncoa7*) and cDC2 (*Sirpa* and *Itgam*) (Fig. 1A; Fig. S1A). In addition, non-classical monocytes, classical monocytes, and various monocyte-derived populations (moDCs) were identified (Fig. 1A; Fig.S1A). One of the most interesting outcomes of this analysis was a very distinct transcriptional separation of cDC1 from other DC subsets, suggesting their unique role in the immune response (Fig. 1A; Fig. S1B). Specifically, when compared to cDC2 and monocyte-progeny cells, the top 10 most expressed genes in cDC1 highlighted immunological functions such as cell recruitment/migration (Fscn1, Ccr7, Ccl22, and Lsp1) (25–28), DC activation and antigen presentation (Tmem123 and Serpinb9) (29, 30), metabolism (Tbc1d4) (31), and T cell activation (Serpinb6b) (Table S1). Differentially expressed gene comparison and pathway analysis revealed that the Jak-STAT signaling pathway and its related genes such as *IL12b* and *Stat4* were predominantly enriched in cDC1 and to a lesser degree *Stat3* compared to other DC and DC-like subsets within the brain (Fig. 1B through D). Notably, *IL12b* and *Stat4*, which play specific roles in Th1 polarization, were predominantly upregulated in cDC1 but not in other types of DC and DC-like cells (Fig. 1D). Moreover, cDC1 notably upregulated *Ccl22*, crucial for T-cell recruitment (Fig. 1B). Taken together, our findings indicate that in a Th1-dominant anti-cryptococcal response, cDC1 has a unique transcriptional signature, hinting of their major role in shaping T-cell responses.

### Batf3, and by extension cDC1, is pivotal for orchestrating effective immune responses against cryptococcal infection, particularly within the brains and lungs

**Fig 2:**
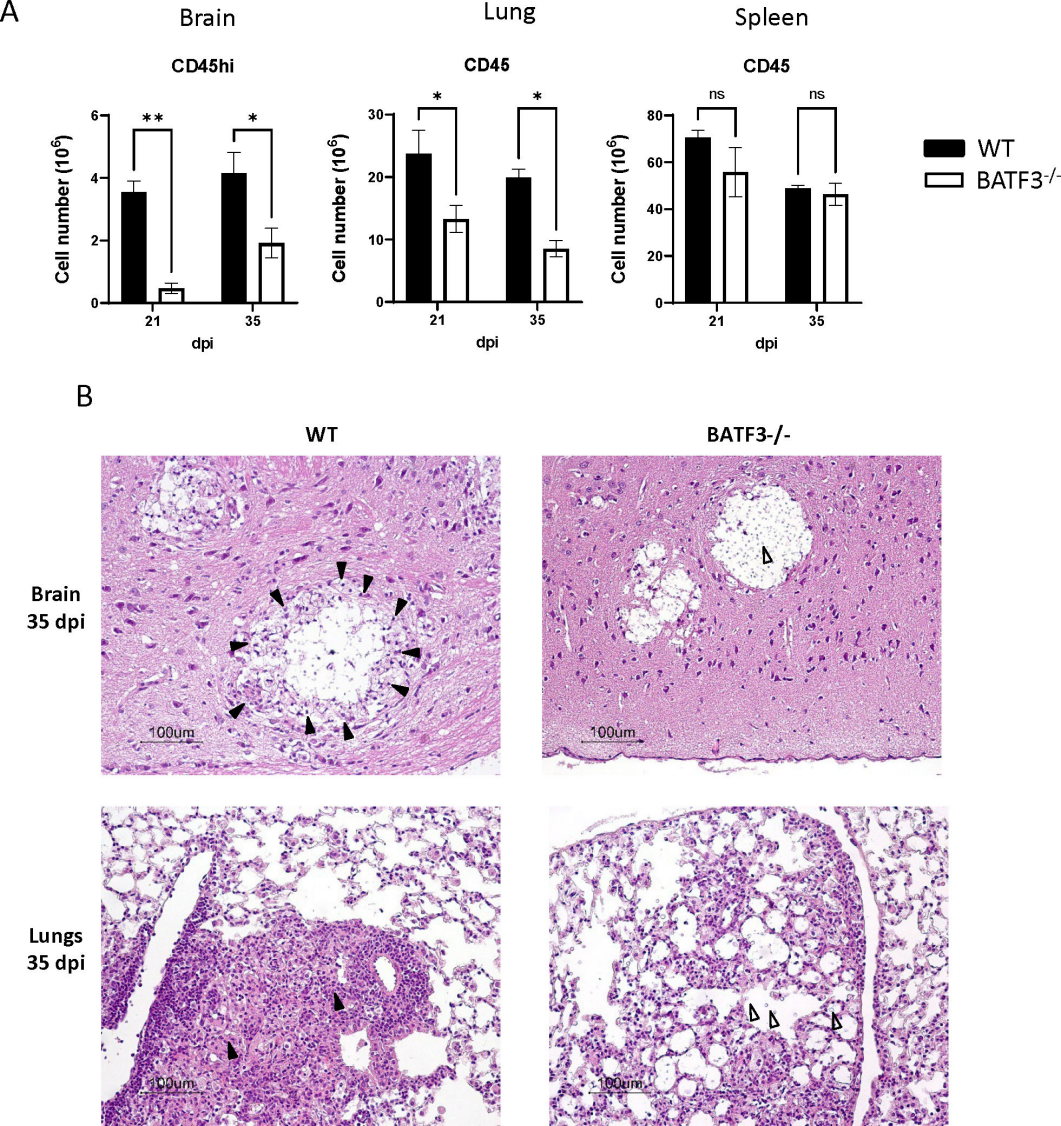
Batf3-dependent cDC1 enhances immune infiltration in the brain and lungs during cryptococcal infection. (**A**) Immune cells from the brain, lungs, and spleens were extracted and evaluated via flow cytometry at 21 and 35 dpi. (**B**) Histological analyses of brain and lung tissues were conducted using H&E staining. Solid arrows point to immune infiltrations surrounding cryptococcal lesions, while blank arrows highlight the extracellular growth of *Cryptococcus neoformans* in the tissues. Data shown are the mean ± standard error of the mean (SEM) from an experiment representative of two independent experiments (*n* > 4). *, *P* < 0.05; **, *P* < 0.01, ns, non-significant. The histology photomicrographs presented are representative samples from three different mice per group across two separate experiments.

To further understand cDC1’s role, we next used Batf3^−/−^ mice, which specifically lack the cDC1 population (14, 32, 33). When comparing inflammation in various organs of infected mice at both 21 and 35 days post-infection (representing peak and late phase of inflammation) (23), we observed markedly reduced CD45+ immune cell infiltration into the brains and lungs of Batf3^−/−^ mice, though not in the spleens (Fig. 2A). Histological evaluations revealed that in wild-type (WT) mice, intense immune infiltrates effectively encircled the cryptococcal lesions in both the brain and lungs. In stark contrast, we observed that Batf3^−/−^ mice lacked the well-developed margin of inflammation around cryptococcal lesions. *C. neoformans* formed prominent, “Swiss cheese”-type lesions where fungus proliferated extensively in extracellular compartments in the brain forming numerous pseudocysts in Batf3^−/−^ mice. In the lungs, Batf3^−/−^ mice no longer contained the fungus within the dense leukocyte infiltrates, but exhibited more diffused immune infiltrations, and *C. neoformans* scattered over larger areas of the lungs, without a defined border (Fig. 2B). Collectively, our observations indicate the indispensable role of Batf3-dependent cDC1 in marshaling a robust immune response, crucial for the effective containment of fungal expansion.

### Role of Batf3/cDC1 in type 1 cytokines production during cryptococcal infection

**Fig 3:**
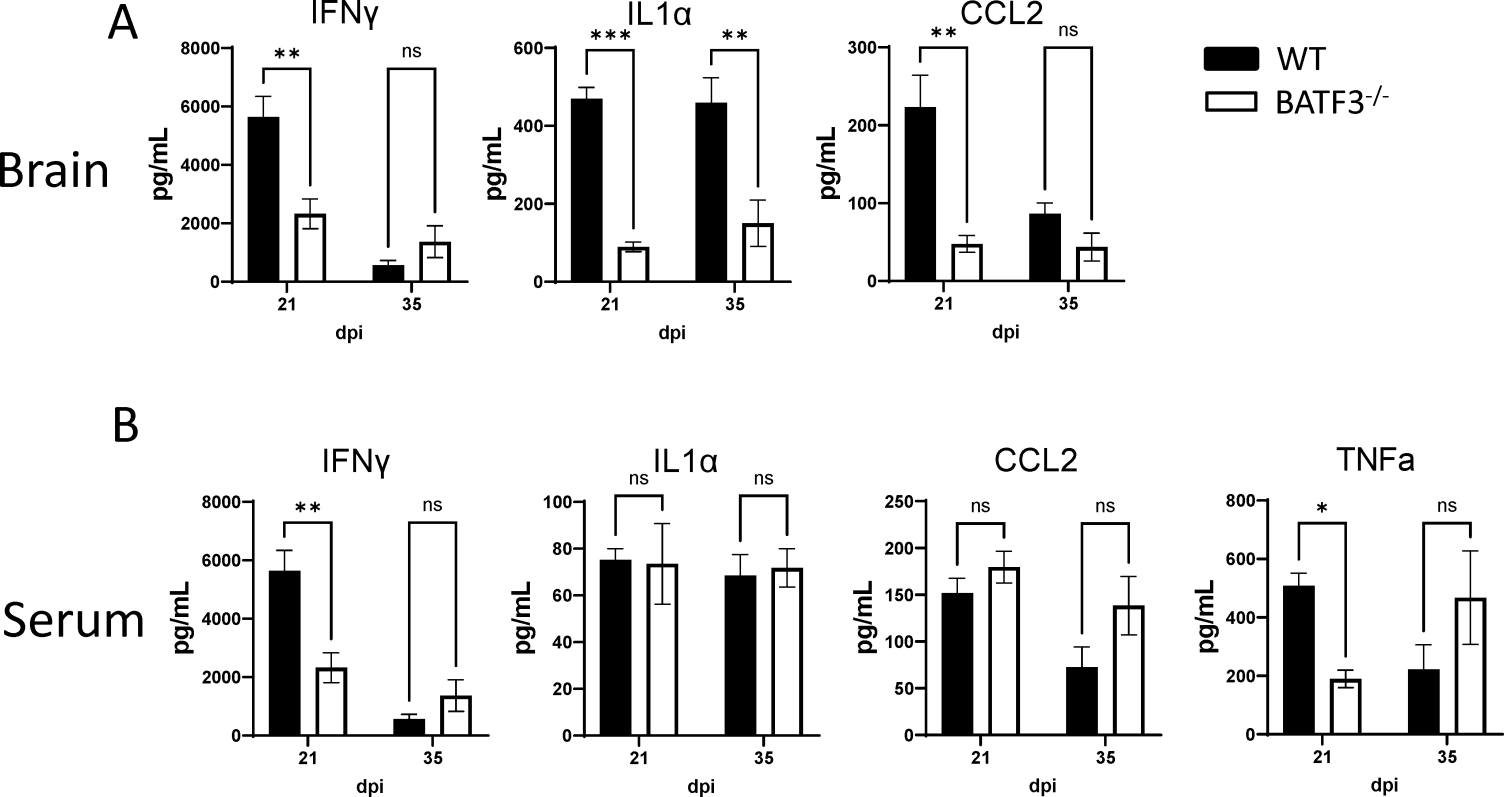
Batf3-dependent cDC1 enhances type 1 cytokine production amid cryptococcal infection. (**A**) Analysis of IFNγ, IL-1α, and CCL2 cytokine levels in brain homogenate at 21 and 35 dpi using the CBA assay. (**B**) Evaluation of IFNγ, IL-1α, CCL2, and TNFα cytokine levels in mouse serum at 21 and 35 dpi via the CBA assay. Data shown are the mean ± standard error of the mean (SEM) from an experiment representative of two independent experiments (*n* > 4). **, *P* < 0.01, ***, *P* < 0.001, ns, non-significant.

Next, we analyzed the cytokine production by WT and Batf3^−/−^ mice during cryptococcal infection. Batf3-deficient mice manifested a marked reduction in the production of IFNγ, IL1α, and CCL2 proteins within the brain compared with WT control mice at both 21 and 35 dpi (Fig. 3A). We extended our examination to their systemic production in the serum. There was a significant decrease in the serum levels of IFNγ in Batf3^−/−^ mice at 21 dpi, while IL1α and CCL2 remained largely unchanged (Fig. 3B). Moreover, serum TNFα levels were found to be reduced in Batf3^−/−^ mice at 21 dpi (Fig. 3B). However, this distinction in IFNγ and TNFα was not observed at 35 dpi, when inflammation recedes (Fig. 3B). Thus, Batf3-dependent cDC1 promotes the production of type 1 cytokines, notably IFNγ and TNFα, which are crucial components of effective defenses against cryptococcal infection (34–36).

### Role of Batf3/cDC1 in Th1 response during cryptococcal infection

**Fig 4:**
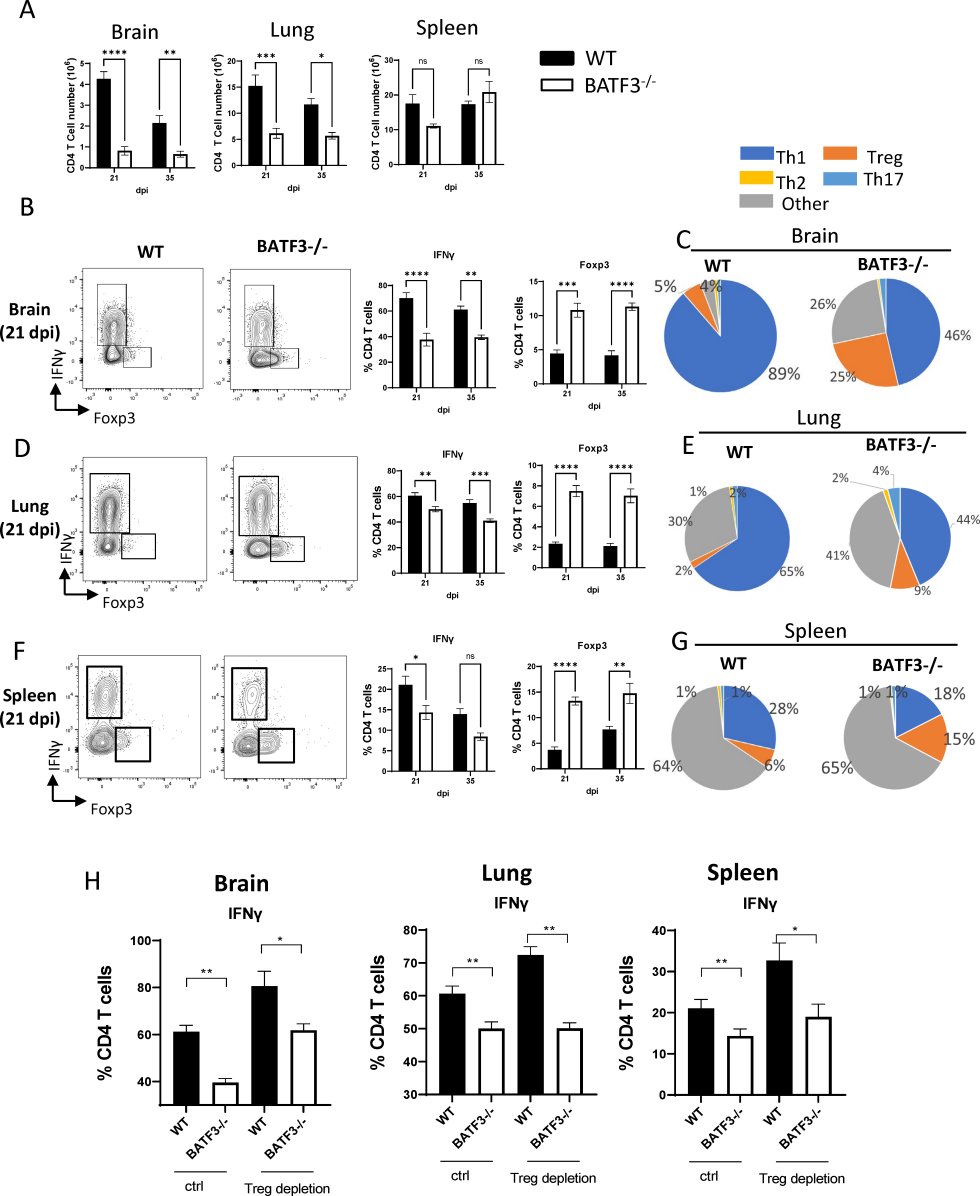
BATF3-dependent cDC1 augments Th1 responses in cryptococcal infection. (**A**) Flow cytometry analysis of the total number of CD4 T cells present in the brain, lungs, and spleens. Intracellular flow cytometry further assessed the expression of IFNγ and Foxp3 within CD4 T cells across the brain (**B**), lungs (**D**), and spleen (**F**). The accompanying pie chart (**C, E, G**) illustrates the frequencies of CD4 T cells categorized as Th1, Th2, Th17, or Treg. (**H**) Batf3^−/−^ mice exhibit reduced Th1 responses, unaffected by Treg depletion. Intracellular flow cytometry was employed to evaluate the expression of IFNγ within CD4 T cells from the brain, lungs, and spleens at 21 dpi. WT and Batf3^−/−^ mice were administered either an anti-CD25 antibody or an isotype control to deplete Tregs. Data shown are the mean ± standard error of the mean (SEM) from an experiment representative of two independent experiments (*n* > 4). *, *P* < 0.05; **, *P* < 0.01, ***, *P* < 0.001, ns, non-significant.

We next investigated the role of Batf3-dependent cDC1 in shaping the CD4 T-cell-mediated immune responses. We found a significant decrease in total CD4 T cell numbers within the brains and lungs of Batf3^−/−^ mice when compared to their WT counterparts, both at 21 and 35 dpi (Fig. 4A). Interestingly, this decline was not evident in the spleens (Fig. 4A). We further identified a marked decrease in the frequency of IFNγ+ CD4 T cells in Batf3^−/−^ mice across all three organs, consistent at both 21 and 35 dpi (Fig. 4B, D and F). Intriguingly, there was also an elevation in the frequency of Foxp3+ CD4 regulatory T cells (Tregs) in the Batf3^−/−^ mice at both 21 and 35 dpi (Fig. 4B, D and F). Finally, Th2 and Th17 cytokine production by CD4 T cells remained comparable in both Batf3^−/−^ and WT mice across all organs (Fig S2). In summary, our findings highlight an integral role of Batf3 cDC1 in shaping Th1-centric immune responses across multiple organs post cryptococcal infection.

Next, we probed if the diminished Th1 response in Batf3^−/−^ mice arose due to elevated Treg cells, known for their ability to inhibit Th1 effector functions (37). To investigate this, we depleted Treg cells using an anti-CD25 antibody (38) and compared IFNγ responses in T cells from the infected WT and Batf3^−/−^ mice. We found with WT mice, Treg depletion led to an increased frequency of IFNγ producing CD4 T cells in all three organs at 21 dpi, consistent with the inhibitory role of Tregs (Fig. 4H). Still, despite Treg depletion, Batf3^−/−^ mice continued to exhibit reduced IFNγ production relative to the WT counterparts in brains, lungs, and spleens at 21 dpi (Fig. 4H). Thus, the suppression of Th1 response in the absence of Batf3 is not primarily caused by the Treg activity.

### CD8 T-cell accumulation but not their IFNγ production is modulated by Batf3/cDC1 during cryptococcal infection

**Fig 5:**
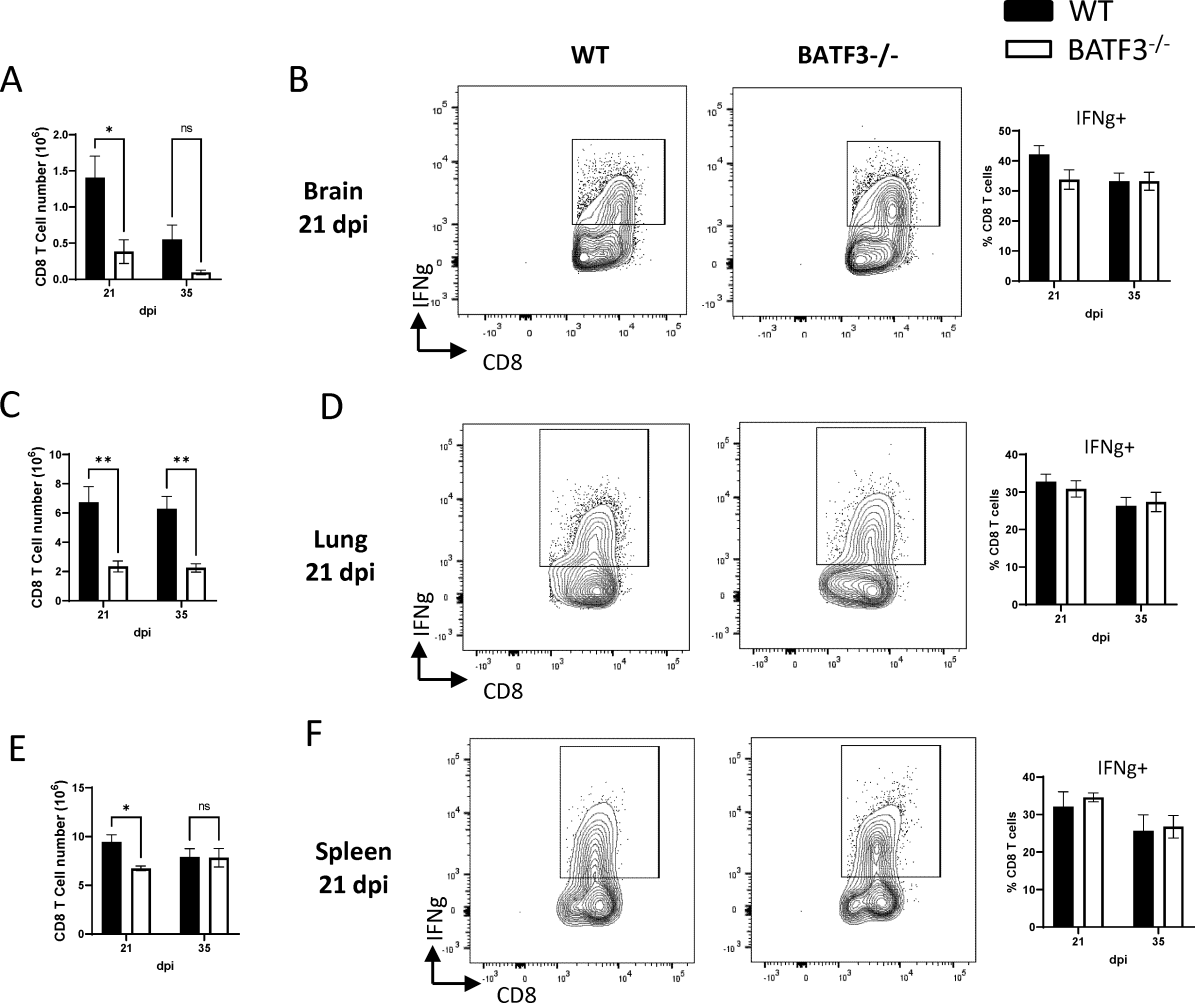
Batf3-dependent cDC1 influences CD8 T-cell accumulation, but not their IFNγ production, during cryptococcal infection. The total number and IFNγ expression within CD8 T cells in the brain (**A and B**), lungs (**C and D**), and spleens (**E and F**) were analyzed by flow cytometry at 21 and 35 dpi. Data shown are the mean ± standard error of the mean (SEM) from an experiment representative of two independent experiments (*n* > 4). *, *P* < 0.05; **, *P* < 0.01. ns, non-significant.

Considering the reported role of cDC1 in cytotoxic T lymphocyte activity (14–16), we explored the potential effect of Batf3/cDC1 deletion on CD8 T-cell dynamics in the context of cryptococcal infection. While a marked decline in CD8 T-cell counts was noted in the brain and lungs (Fig. 5A and C), CD8+ T-cell numbers remained relatively consistent in the spleens (Fig. 5E). Despite the effect on CD8 T-cell accumulation in peripheral organs, we found no effect of Batf3 on their IFNγ production in all three organs (Fig. 5B, D and F). This suggests that Batf3 and by extension, cDC1 exert influence on CD8 T-cell accumulation, without regulating their Th1-polarization in this infection model.

### Classical activation of phagocytes and effective fungal control during cryptococcal infection are linked to the presence of Batf3/cDC1

**Fig 6:**
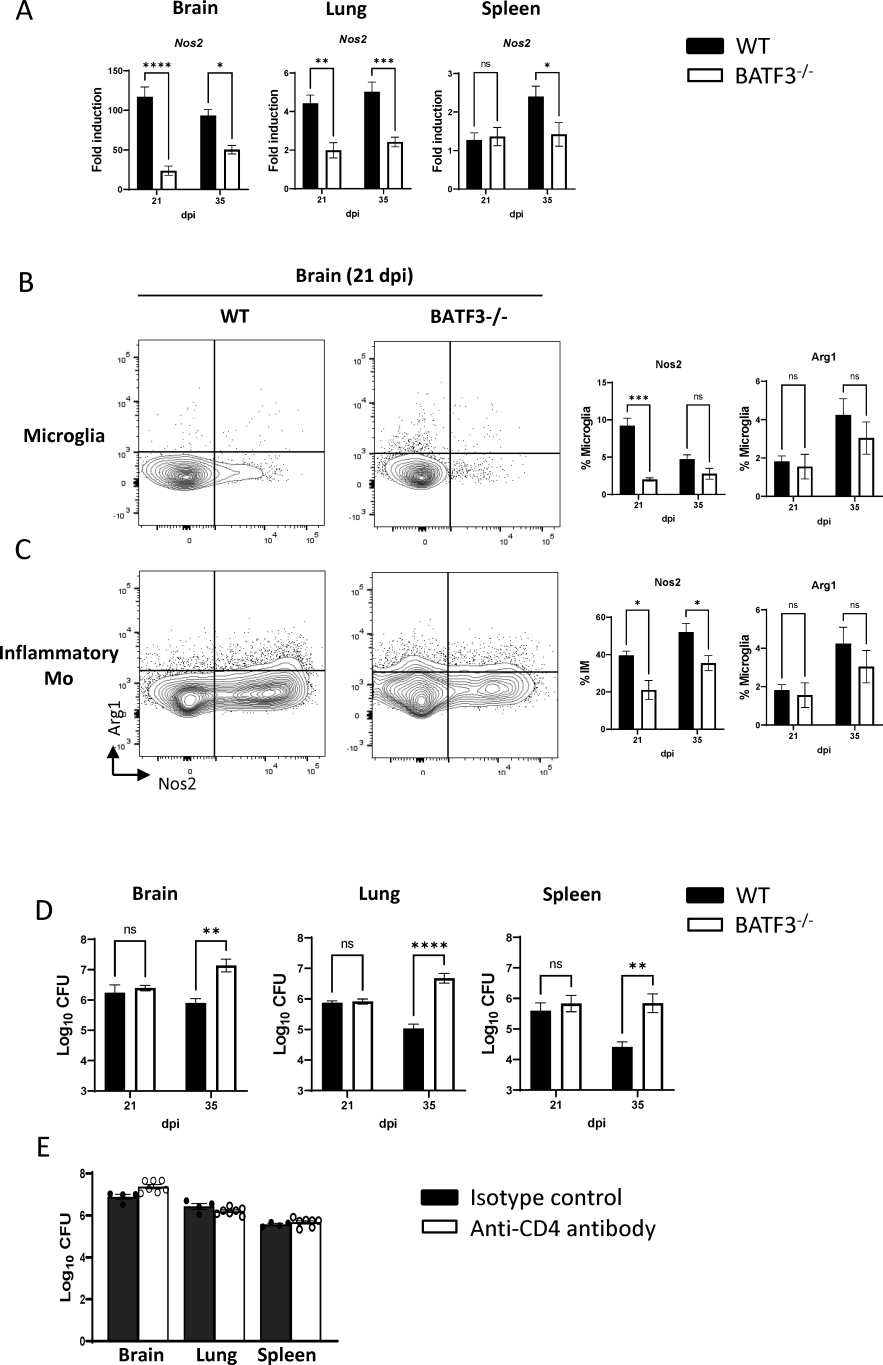
Batf3-dependent cDC1 fosters classical activation of phagocytes and enhances fungal containment during cryptococcal infection. (**A**) Analysis of *Nos2* gene expression in brain, lung, and spleen homogenates was analyzed by qPCR at both 21 and 35 dpi. (**B**) Production of NOS2 and ARG1 proteins by microglia and CD11b Ly6c inflammatory monocytes was evaluated using intracellular flow cytometry at 21 and 35 dpi. (**D**) Analysis of fungal load in the brain, lungs, and spleens was conducted at 21 and 35 dpi. (**E**) Fungal burden in the brain, lungs, and spleens from Batf3^−/−^ mice, which were administered either an isotype control or an anti-CD4 depletion antibody, was assessed at 35 dpi. Data shown are the mean ± standard error of the mean (SEM) from an experiment representative of two independent experiments (*n* > 4). *, *P* < 0.05; **, *P* < 0.01. ***, *P* < 0.001, ns, non-significant.

To further understand the implications of Batf3-dependent cDC1, we probed their potential impact on the host’s ability to effectively combat fungal infection. We first examined the transcript level of the *Nos2* (inducible nitric oxide synthase 2), a widely used marker of classical macrophage activation and fungicidal macrophages (39–41). We found a sharp decline in *Nos2* gene expression in the lungs, brains, and spleens of Batf3^−/−^ mice in contrast to their WT counterparts (Fig. 6A), which translated into reduced NOS2 protein production in both microglia and infiltrating inflammatory monocytes in the brain (Fig. 6B and C). Consistent with the neutral effect of cDC1 on Th2 responses (42), there was no significant change in the expression of Arg1 (Fig. 6B and C), representative of non-protective alternative macrophage activation, driven by Th2 cytokines. Since Th1 response, chiefly IFNγ, is the driver antifungal capabilities of phagocytes, we predicted that Batf3^−/−^ deletion would affect fungal clearance. In line with this, we found a marked contrast in the fungal clearance dynamics: while WT mice demonstrated consistent fungal clearance over time in the lungs, brains, and spleens, the Batf3^−/−^ mice exhibited either a surge in fungal growth (the lungs and brain) or stagnation in clearance in the spleens at 35 dpi (Fig. 6D). Interestingly, while Batf3^−/−^ mice display increased fungal burden, the survival rates and disease progression evaluated using a murine coma and behavior score are not significantly different from WT mice (Fig S3), indicating that different mechanisms likely contribute to the disease progression between Batf3^−/−^ and WT mice. Furthermore, we rigorously depleted the residual CD4 T cells in Batf3^−/−^ mice using an anti-CD4 antibody to examine their remaining potential to impact fungal control. Upon CD4 T-cell depletion, there was no discernible further decrease in fungal control by 35 dpi (Fig. 6E). Collectively, our findings emphasize the indispensable role of Batf3-dependent cDC1 for proper generation of the Th1-arm of the immune response thereby ensuring effective anti-cryptococcal defenses.

## DISCUSSION

The interplay between host immune responses and fungal pathogens remains an important focus in understanding infectious diseases, especially given their rising global burden. In this study, we investigated the role of Batf3-dependent cDC1 in shaping host defense against cryptococcal infection. In a murine model that mounts effective fungal control, scRNA seq revealed that Batf3-dependent cDC1 displays a unique mRNA signature with highly pronounced transcripts for T-cell recruitment and Th1 polarization pathways. Our results further suggest the pivotal roles of cDC1 in generating protective Th1 CD4 T-cell response in this model. Deficiency in Batf3/cDC1 resulted in a dramatic reduction in total immune infiltrates, type 1 cytokines’ production, as well as CD4 accumulations and their IFNγ cytokine production in multiple organs. Mice lacking Batf3/cDC1 exhibited a profound reduction in fungicidal activity by phagocytes and a severe compromise in their ability to control the fungal pathogen. In conclusion, our study provides strong support that Batf3-dependent cDC1 plays a pivotal role in orchestrating protective Th1 response and ensuring robust fungal clearance during cryptococcal infection.

Host defense against cryptococcal infection is heavily dependent on the T-cell-mediated immune responses (6, 35, 43). For our growing understanding of DC’s role in Th1 responses during cryptococcal infection, it is crucial to recognize their functional and phenotypic diversity (12, 44). Recent initiatives have unified naming conventions based on ontogeny, leading to a shared understanding across research communities about comparable DC subsets in both mice and humans (11). Notably, cDC1s and cDC2s, which stem from common DC progenitors in the bone marrow, are prevalent in both lymphoid and non-lymphoid tissues (11). Conversely, monocyte-derived cells (moDCs) differ fundamentally from cDCs, given their origin from monocytes. One critical research gap is understanding of the specific interactions between these DC subsets and the cryptococcal pathogen. While a study pinpointed the CD11b + cDC2 subset as a key driver of the non-protective Th2 immune response during cryptococcal infection (42), the mechanisms by which DC subsets initiate and modulate the protective Th1 response remain unclear. There is a recognized link between the chemokine receptor 2 (CCR2) dependent moDC and a robust Th1 response (45–47). However, in a strongly Th2-biased pulmonary infection model, this very same cell subset promotes undesirable Th2 and opposes fungal clearance (48). Taken together, these conflicting results suggest that moDC may act as an amplifier of pre-polarized T cells, rather than performing the antigen transport to lymph nodes and priming of naive T cells.

Even less is known about cDC1 in cryptococcal infections, but their proficiency in producing IL12 in multiple inflammatory settings has been reported (17, 19, 49, 50). In our earlier research, where transient TNFα-depletion was associated with lasting dysregulation of immune response and failure to generate robust Th1 responses, we observed a significant reduction of cDC1 accumulation into lung draining nodes, which correlated with diminished Th1/Th17 responses (51), but the role of cDC1 remains unclear in the context of cryptococcal infection. cDC1 differentiation is orchestrated by a series of tightly regulated transcription factors, chiefly Batf3 (33). In mice, a genetic deletion of Batf3 specifically leads to the significant loss of cDC1, and this tool has been utilized to explore the significance of cDC1 in various conditions (49). In our current research, we aimed to determine the role of cDC1 in guiding host defense against cryptococcal infection. Our findings were striking: the absence of Batf3/cDC1 resulted in a stark decrease in Th1 response/IFNγ-producing CD4 T cells, subsequent weakening of macrophage fungal defense, and resultant rampant fungal growth in multiple organs of mice, highlighting its critical roles in host defense against cryptococcal infections. Our study also further reinforces previously reported findings on Batf3/cDC1’s neutral position regarding type 2 responses during cryptococcal infection (42). Regarding the T-cell recruitment to the infected organs, we see the strongest effect of cDC1 in the brain and lungs but not in the spleen. This outcome suggests that the cDC1 may have a powerful effect on T-cell recruitment to the peripherally infected organs, without affecting their proliferation rate or steady state status in the spleen, a secondary lymphoid organ. The significance of cDC1 in T-cell trafficking to the peripheral target organs, including the brain, may be particularly important, considering that meningoencephalitis is the most fatal manifestation of cryptococcal infection.

While our data support that cDC1 promote both polarization and recruitment of T cells in our disseminated infection model; however, a small remaining number of T cells still producing IFNγ are detected in our leukocyte isolates from Batf3^−/−^ mice. It is quite likely that other DC subsets detected in our scRNAseq analysis (Fig. 1), can to a degree, compensate for the lack of cDC1. Yet, their capacity to augment the Th1 response proves to be notably inferior to cDC1, making them less effective as replacements in the absence of cDC1. The significance of Batf3/cDC1 in this context becomes even clearer from our CD4 T-cell depletion experiments: the removal of remaining CD4 T cells in Batf3^−/−^ mice did not further cause any additional expansion of fungal organisms. The outcome of meningoencephalitis is delicately balanced between fungal proliferation and host inflammation. Indeed, while Batf3-deficient mice display increased fungal burden, their survival rates and disease progression are not significantly different from WT mice. The most likely explanation for why the expected increase in mouse mortality has not occurred in Batf3^−/−^ mice is that the detrimental effect of increased fungal burden was counterbalanced by the alleviation of inflammatory immunopathology in the brain reported in their WT counterparts (Fig. 3A) (23, 24).

The use of Batf3^−/−^ mice has provided invaluable insights into the functions of cDC1 in various settings (14, 18, 19, 22, 32). However, Batf3 is also expressed in other cells. Although Batf3 deficiency does not inherently influence CD8+ T-cell differentiation or inflammatory cytokine activity, it does interfere with the memory formation of CD8+ T cells post-infection (52). We observed elevated Treg frequency in Batf3^−/−^ mice, in line with previous findings highlighting Batf3’s intrinsic association with Tregs (53). Yet, our data decisively show that the suppressed Th1 response during cryptococcal infection cannot be attributed to this increased Treg activity. Significantly, the intrinsic absence of Batf3 in T cells does not create notable disruptions in the development or cytokine output of other helper T cells, like Th1, Th2, and Th17 in *ex vivo* stimulation experiments (14). However, the potential role of other myeloid populations such as microglia who also to a lesser extent express the *Batf3* gene (54) cannot be entirely ruled out, and further studies are needed to clarify this point.

The function of cDC1 in directing the responses of CD4 or CD8 T cells has profound effects on the subsequent antifungal capabilities of mononuclear phagocytic cells. A distinguishing trait of robust host defense against fungal invasions is the classical activation of these cells (55), of which NOS2 expression serves as a key indicator. Remarkably, the absence of Batf3-dependent cDC1 comes with a significant drop in *Nos2* transcript and protein expression in crucial organs, highlighting their pivotal role in the classical activation pathway needed for fungal clearance. Our data support that the defect in classical activation of recruited inflammatory monocytes and resident microglia is most likely driven by the weaker Th1 response in terms of cell numbers and IFNγ production. In support of this view is the unaltered Arg1 expression in phagocytes from the infected Batf3^−/−^ mice, which aligns with the unaltered type 2 cytokine production from CD4 T cells and our previous work which defined the chief role of IFNγ and IL-4 in macrophage polarization during cryptococcal infection (56).

In conclusion, our study emphasizes the indispensable role of Batf3 and by extension cDC1 in orchestrating Th1 responses and combatting cryptococcal infection. These findings strongly suggest that a defect in Batf3/cDC1 signaling could be a possible explanation for a subset of cryptococcal infections that spontaneously occur in previously healthy patients and that cDC1 likely offers a promising avenue to bolster host defenses against this lethal fungal adversary.

## MATERIALS AND METHODS

### Mice

Male and female C57BL/6J mice were provided by Jackson Laboratories (Bar Harbor, ME). BATF3^−/−^ mice (strain 013755, The Jackson Laboratory, USA) were housed in specific pathogen-free conditions at the VA Ann Arbor Healthcare System’s Animal Care Facility. Mice were infected at 8–12  weeks old and were humanely euthanized at the time of data collection by CO_2_ inhalation. All experiments were approved under protocol 1408-004 of the Veterans Affairs Institutional Animal Care and Use Committee and were performed according to NIH guidelines and the Guide for the Care and Use of Laboratory Animals. Data were obtained from both male and female mice, with no significant differences in CFU, murine coma and behavioral scale (MCBS), survival, or brain cell accumulation between sexes observed during *C. neoformans* brain infection.

### Cryptococcal culture and infection

*C. neoformans* strain ATCC 24067 (American Type Culture Collection, Manassas, VA) was recovered from 10% glycerol frozen stocks and grown for 96  h at 37°C in Sabouraud dextrose broth (1% neopeptone, 2% dextrose; Difco, Detroit, MI) on a shaker. The cultures were then washed by several steps of centrifugation and re-suspension in phosphate-buffered saline (PBS). The cells were counted with a hemocytometer and the suspension concentration was diluted to 5  ×  10^6^ cells/mL before infection. Mice were inoculated with 10^6^ organisms (in 200 µL PBS) under inhaled isoflurane anesthesia by retro-orbital intravenous injection. The concentration of viable fungi in the inoculum was confirmed by plating serial dilutions on Sabouraud dextrose agar.

### Fungal burdens

The total fungal burden was quantified per organ. Brains and spleens were homogenized and serially diluted with sterile distilled water. About 10 µL of aliquots of each dilution level of each sample was plated in duplicate on Sabouraud dextrose agar. CFU counts for each sample were determined after a 48-h incubation at room temperature.

### Brain leukocyte isolation

Leukocytes in the brain were isolated as previously described (57). In brief, mice were euthanized with CO_2_ and then perfused with PBS to remove circulating red blood cells and leukocytes from the brain. The brains were then removed and placed in 5  mL of digest medium [RPMI 1640 with 5% fetal bovine serum (FBS), 25  mM HEPES, GlutaMAX, penicillin-streptomycin, nonessential amino acids, sodium pyruvate, and beta-mercaptoethanol, collagenase (50  µg/mL; Roche), and DNase (100 U/mL; Worthington)] in gentleMACs C tubes. The tissue was minced using scissors and then homogenized with a gentleMACs Dissociator (Miltenyi). The homogenate was washed with RPMI 1640 and passed through a 70-µm cell strainer to remove large debris. A discontinuous 30%/70% Percoll (GE Healthcare) gradient was created and microglia and brain-infiltrating leukocytes (BILs) were recovered from the interface (leaving behind cell debris, myelin, and neurons). Isolated cells were washed two times in PBS. Live cells were counted on a hemocytometer in trypan blue (Sigma) to determine the total cell number.

### Lung leukocytes isolation

Lung leukocytes were isolated as previously described (26). Briefly, the lungs from each mouse were excised following CO_2_ euthanasia and PBS perfusion and then washed in RPMI 1640. Lungs were minced and then homogenized with a gentleMACS Dissociator in gentleMACs C Tubes. Homogenate was incubated at 37°C for 35 min in 5 mL/mouse digestion buffer [RPMI 1640, 5% FBS, penicillin and streptomycin (Invitrogen, Grand Island, NY); 1 mg/mL collagenase A (Roche Diagnostics, Indianapolis, IN); and 30 µg/mL DNase I (Sigma, St. Louis, MO)] and further dissociated by another round of gentleMACS homogenization. The lung digest mixture was centrifuged and 3 mL NH4Cl buffer (0.829% NH4Cl, 0.1% KHCO3, and 0.0372% Na2EDTA, pH 7.4) was added over the pellet to lyse erythrocytes for 3 min, after which a threefold excess of RPMI 1640 was added to prevent leukocyte lysis. The cells were re-suspended, dispersed by repeated drawings into a syringe, and filtered through a sterile 100 µm nylon screen (Nitex, Kansas City, MO). The filtrate was centrifuged in the presence of 20% Percoll (Sigma) at 1,500 × *g* for 30 min with no brake to separate cell debris and epithelial cells from leukocytes. Leukocyte pellets were re-suspended in complete media (10% FBS in RPMI 1640), diluted in trypan blue, and placed on a hemacytometer for live-cell counting.

### Splenocyte isolation

Spleen leukocytes were isolated following perfusion with PBS. Immediately after excision, spleens were placed in PBS and kept on ice for the remainder of the harvest procedure. Spleens were then transferred to a 70-µm cell strainer pre-rinsed with 5 mL of RPMI 1640 atop a 50-mL conical vial and thoroughly broken up with a syringe plunger. The cell strainer was then rinsed with 5 mL of RPMI 1640 to transfer residual cells into the 50 mL conical vial. Spleen cells were then pelleted via centrifugation (1,200 rpm for 10 min at room temperature with brake on high) and re-suspended in 5 mL NH4Cl buffer (0.829% NH4Cl, 0.1% KHCO3, and 0.0372% Na2EDTA, pH 7.4) for 5 min of erythrocyte lysis on ice. Lysis was stopped by the addition of 10 mL RPMI and cells were again pelleted by centrifugation. After re-suspension in complete media (10% FBS in RPMI 1640), cells were diluted 100-fold in Trypan blue and counted on a hemacytometer to determine live-cell counts.

### CD4 T cells and Treg depletion

Mice underwent CD4+ T-cell depletion through intraperitoneal administration of the GK1.5 monoclonal anti-CD4 antibody (300 µg) on days −2, 0, and 5 post-infection, followed by weekly doses. Controls were treated with an equivalent dose of the anti-keyhole limpet hemocyanin (anti-KLH) isotype antibody (LTF-2; 300 µg). For the depletion of regulatory T cells, we administered the anti-CD25 antibody (PC-61.5.3). An initial dose of 200 µg was given on day 0, followed by another dose on day 5 post-infection, and subsequent weekly doses of 100 µg thereafter. All antibodies, of *in vivo* grade purity, were sourced from BioXCell, West Lebanon, NH.

### Flow cytometry

Isolated cells were stained with fixable live/dead dye (Life Technologies), blocked with anti-CD16/32, and stained with fluorescently labeled antibodies for CD45 (30-F11), CD3 (145-2C11), CD4 (GK1.5), CD8 (53-6.7), CD11b (M1/70), CD11c (N418), Ly6C (HK1.4), F4/80 (BM8), CD64 (X54-5/7.1), XCR1 (ZET), SIRPα (P84), and/or major histocompatibility complex class II (MHCII, M5/114.15.2).

For assessment of intracellular iNOS and ARG1 production by myeloid cells, the cells were fixed and permeabilized with 2% formaldehyde for 30 min following extracellular staining. The cells were then intracellularly stained with anti-iNOS (CXNFT) and anti-ARG1 (IC5868P) antibodies in the permeabilization buffer.

To assess intracellular cytokine production by T cells, stimulation was performed by incubating the cells for 6 h with phorbol myristate acetate and ionomycin with brefeldin A and monensin present for the final 4 h. Extracellular staining was performed, followed by incubation in fixation/permeabilization buffer, after which cells were stained for FoxP3 and IFN- γ in the fixation/permeabilization buffer. Fluorescence minus one (FMO) controls were used in all experiments. Data were collected using either an LSRII flow cytometer (BD Biosciences) or LSRFortessa flow cytometer (BD Biosciences) and were analyzed with FlowJo software (TreeStar).

### Cytokine expression

Blood samples were obtained from recently euthanized mice by severance of the vena cava before lung excision. Samples were allowed to clot and then centrifuged for 20 min at 10,000 rpm to produce a serum supernatant. Whole brain homogenate was obtained by mincing brains with scissors after PBS perfusion and then homogenizing with a gentleMACs Dissociator (Miltenyi) in digest medium [RPMI 1640 with 5% fetal bovine serum (FBS), 25  mM HEPES, GlutaMAX, penicillin-streptomycin, nonessential amino acids, sodium pyruvate, and beta-mercaptoethanol, collagenase (50  µg/mL; Roche), and DNase (100 U/mL; Worthington)] in gentleMACs C tubes. Cytokine levels in the supernatants of whole-brain homogenate or serum were measured with LegendPlex cytometric bead assays (BioLegend) per manufacturer instructions.

### Mouse survival and murine coma and behavioral scale

In mortality studies, animals were euthanized when they lost 20% body weight, had persistent cranial swelling, and/or developed neurological symptoms. A MCBS was used to assess the overall physical and neurological condition of infected mice as described previously (23). Briefly, mice were scored using a scale of 0–3 for exploration, balance, gait, body posture, coat condition, grip strength, reflexes (body, neck, pinna, and footpad reflexes), and response to visual stimuli. Lower scores reflect more pronounced symptoms.

### Single-cell RNA library preparation, sequencing, and analysis

Brain leukocytes from days 0, 7, 14, 21, and 28 post-infection were isolated using the aforementioned methods. CD45+ immune cells were enriched following the manufacturer’s protocol for CD45 MicroBeads, mouse (Miltenyi Biotec). For all samples, cell viability was above 90%, as assessed by trypan blue staining. Using the Chromium Next GEM Single Cell 3ʹ Reagent Kit v3.1 (10× Genomics), we constructed scRNA-seq libraries. Briefly, cell suspensions (700–1,200 cells/µL) were loaded onto a Chromium controller to create single-cell GEMs. Libraries were then assembled as per the manufacturer’s guidelines and sequenced on an Illumina Novaseq at the University of Michigan’s Advanced Genomics Core, following 10× Genomics' recommended cycling. The data were aligned to the mouse mm10 genome using the cellranger pipeline (v6.0.0). Initial single-cell expression matrices from cellranger underwent quality control: cells with <500 UMI counts, <200 detected genes, or >20% mitochondrial genes were excluded. We also utilized DoubletFinder to remove doublets formed from distinct cells, with a predicted doublet rate set at 0.075. We utilized Seurat for single-cell integration and clustering (58). Each cell’s gene counts were normalized using the LogNormalize method. This divides the gene counts by that cell’s total counts, then multiplies by the scale.factor. Subsequently, these normalized counts underwent a natural log transformation with the log1p function. For clustering, the top 2,000 variable genes were identified using the FindVariableFeatures function. We set dimension reduction to 50 and resolution at 0.6 for clustering. Cell types within clusters were ascertained based on the prevalent cell type. Subsequently, based on gene expression profiles, monocytes, and DCs were isolated and re-clustered with a dimension reduction of 50 and a resolution of 0.3. We identified feature genes and pathways for cDC1s by contrasting them with other DCs and monocyte types. The GSEA analysis was conducted using the fgsea package (59). The data are deposited to GEO (GSE253846).

### Statistical analysis

Statistical analysis was performed with GraphPad Prism 9 software (Dotmatics), using Student’s *t* test or analysis of variance and Tukey’s post hoc test for multiple comparisons. Statistical significance in figures (in graphs and corners of flow cytometry plots) is indicated by asterisks as follows: *, *P* < 0.05; **, *P* < 0.01; ***, *P* < 0.001; ****, *P* < 0.0001.

## SUPPLEMENTAL MATERIAL

Supplemental figures mbio.02853-23-s0001.pdf Figures S1-S3.

Table S1 mbio.02853-23-s0002.csv Differentially expressed genes in cDC1.

ASM does not own the copyrights to Supplemental Material that may be linked to, or accessed through, an article. The authors have granted ASM a non-exclusive, world-wide license to publish the Supplemental Material files. Please contact the corresponding author directly for reuse.
