# Supplementary material for: Batf3-dependent orchestration of the robust Th1 responses and fungal control during cryptococcal infection, the role of cDC1
Source: mBio. 2024 Feb 13;15(3):e02853-23. doi: 10.1128/mbio.02853-23 (PMC10936214; doi:10.1128/mbio.02853-23)
Supplement: Supplemental figures — Figures S1-S3. [file mbio.02853-23-s0001.pdf]

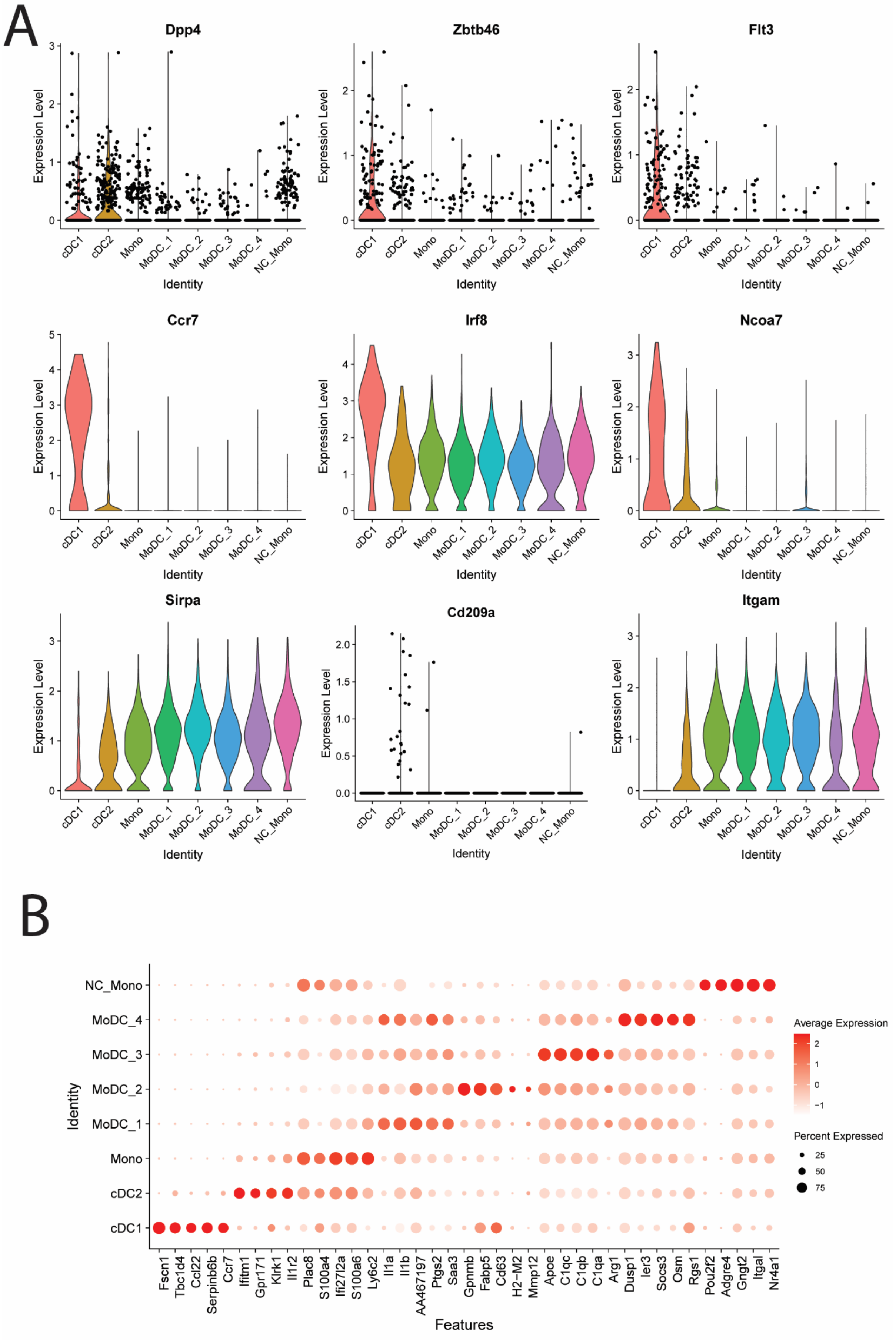

Fig S1. Distinctive DC Marker Gene Expressions.
A) Marker genes delineating DCs, cDC1, and cDC2 are presented.
B) The top 5 representative genes for each cluster.

Fig S2

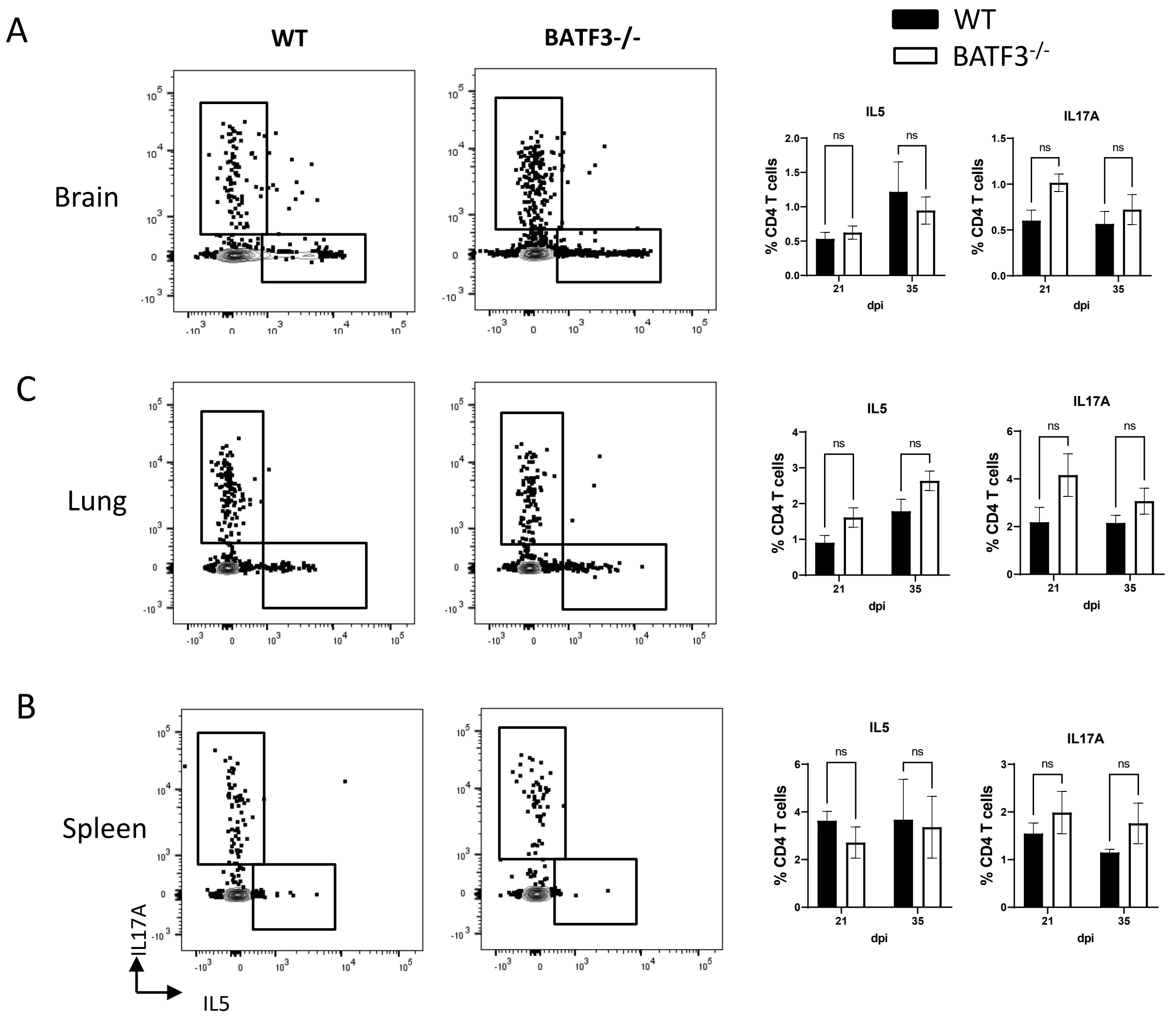

Fig S2. Batf3-Dependent cDC1 Lacks Influence on Th2 and Th17 Responses in Disseminated Cryptococcal Infection. Intracellular flow cytometry analysis detailing the expression of IL5 and IL17A within CD4 T cells from the brain, lungs, and spleen. Data shown are the mean ± standard error of the mean (SEM) from an experiment representative of two independent experiments (n > 4). ns, non-significant.

Fig S3

A B

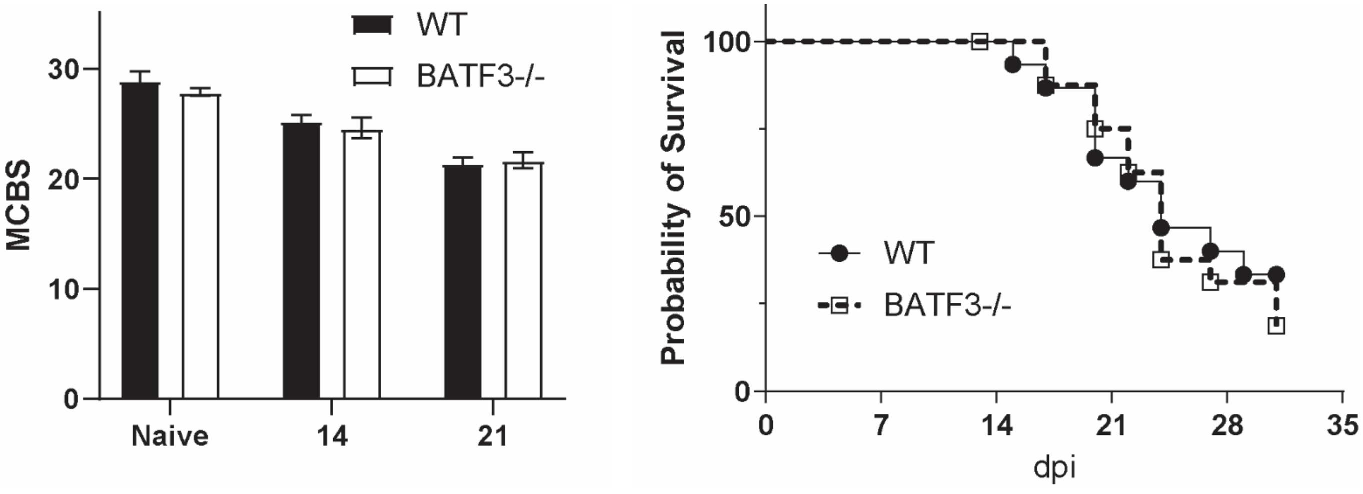

Fig S3. Survival and MCBS are not significantly different between Batf3-/- and WT mice, despite difference in fungal burden. A) MCBS at d0, d14, and d21 post-infection for Batf3-/- and WT mice. B) Survival curves for Batf3-/- and WT mice. Data shown are the mean ± standard error of the mean (SEM) from an experiment representative of two independent experiments (n > 8).
